# Alginate Biofunctional Films Modified with Melanin from Watermelon Seeds and Zinc Oxide/Silver Nanoparticles

**DOI:** 10.3390/ma15072381

**Published:** 2022-03-23

**Authors:** Łukasz Łopusiewicz, Szymon Macieja, Mariusz Śliwiński, Artur Bartkowiak, Swarup Roy, Peter Sobolewski

**Affiliations:** 1 Center of Bioimmobilisation and Innovative Packaging Materials, Faculty of Food Sciences and Fisheries, West Pomeranian University of Technology Szczecin, Janickiego 35, 71-270 Szczecin, Poland; ms40205@zut.edu.pl (S.M.); artur-bartkowiak@zut.edu.pl (A.B.); 2 Dairy Industry Innovation Institute Ltd., Kormoranów 1, 11-700 Mrągowo, Poland; mariusz.sliwinski@iipm.pl; 3 School of Bioengineering and Food Technology, Shoolini University, Solan 173229, Himachal Pradesh, India; swaruproy2013@gmail.com; 4 Department of Polymer and Biomaterials Science, Faculty of Chemical Technology and Engineering, West Pomeranian University of Technology Szczecin 45 Piastów Ave, 70-311 Szczecin, Poland; piotr.sobolewski@zut.edu.pl

**Keywords:** melanin, watermelon, alginate, bioactive films, nanoparticles

## Abstract

Bioactive films find more and more applications in various industries, including packaging and biomedicine. This work describes the preparation, characterization and physicochemical, antioxidant and antimicrobial properties of alginate films modified with melanin from watermelon (*Citrullus lanatus*) seeds at concentrations of 0.10%, 0.25% and 0.50% *w*/*w* and with silver and zinc oxide nanoparticles (10 mM film casting solutions for both metal nanoparticles). Melanin served as the active ingredient of the film and as a nanoparticle stabilizer. The additives affected the color, antioxidant (~90% ABTS and DPPH radicals scavenging for all melanin modified films) and antimicrobial activity (up to 4 mm grow inhibition zones of *E. coli* and *S. aureus* for both zinc oxide and silver nanoparticles), mechanical (silver nanoparticles addition effected two-fold higher tensile strength), thermal and barrier properties for water and UV-vis radiation. The addition of ZnONP resulted in improved UV barrier properties while maintaining good visible light transmittance, whereas AgNP resulted in almost complete UV barrier and reduced visible light transmittance of the obtained films. What is more, the obtained films did not have an adverse effect on cell viability in cytotoxicity screening. These films may have potential applications in food packaging or biomedical applications.

## 1. Introduction

During the transport and storage of food products, it is important to ensure that the product is protected from any negative physical, chemical, or microbiological factors. In this way, packaging should delay food spoilage and slow down the loss of beneficial properties of the processed product, including both visual and nutritional qualities [1]. For example, wax coatings applied to the surface of fruits to extend their shelf life were the first materials used as a coating on food products [2]. In low-income countries, the main share of the total waste is those of organic origin, while in high-income countries, inorganic waste is the most common [3]. Potentially, some of these organic wastes (including agro-industrial by-products) can be used as a source of bioactive compounds such as phenolic compounds, dietary fiber, polysaccharides, vitamins, carotenoids, pigments and oils [4].

Plastics, due to their ease of processing and relatively low cost of raw materials, have become one of the most widely used materials in virtually all industries. Their annual production exceeds 300 million tons worldwide [5]. However, plastics present a problem with regard to waste management: much of plastic waste consists of packaging waste, of which up to 60% is food packaging. Furthermore, the majority of plastic waste (approximately 80%) ends up in landfills or pollutes land or water reservoirs, thus affecting entire ecosystems, from plants through to animals and humans [6,7,8]. Additionally, plastic production and disposal generate large amounts of greenhouse gases, which also has a significant negative impact on the environment. Therefore, finding less harmful alternatives to conventional plastics, such as renewable or biodegradable materials, is important to reduce the harmful impact of packaging on ecosystems [9].

One promising strategy involves biopolymers, polymers of natural origin: usually proteins or polysaccharides obtained from plants, bacteria, fungi, animals or seaweed. As biopolymers have extremely diverse properties, they can find applications in many different industries, including use in packaging, for example, of medical equipment, agricultural products, and other commodities [10,11].

Polysaccharides are a class of biopolymers that are particular abundant, including cellulose, chitin, xanthan gum, starch, carrageenan, and alginate [12]. Alginates are naturally occurring polymers consisting of linear copolymers of D-mannuronic and L-guluronic acids, linked by β-1,4-glycosidic bonds. Depending on the source, the two monomers can differ in proportion and be arranged in different, irregular patterns—these structural aspects affect the properties of alginate-based materials [13]. Example sources include seaweeds, mainly algae (*Laminaria hyperborea, L. digitata, L. japonica, Ascophyllum nodosum,* or *Macrocystis pyrifera*), whose alginic acid is a component of cell walls, or bacterial biosynthesis using *Azotobacter* and *Pseudomonas* bacteria [14]. Alginates have found applications in the textile, food, pharmaceutical, and chemical industries [15]. Importantly, the alginate backbone includes many free hydroxyl and carboxyl groups, making them highly amenable to modification and functionalization, using techniques such as oxidation, esterification and amidation. In this way it is possible to significantly influence the material properties, both physicochemical (such as solubility and affinity for water), as well as biological [16].

Melanins are a group of high molecular weight pigments, formed by the oxidative polymerization of indole and phenolic compounds [17]. In addition to imparting color to living organisms, melanins are also responsible for free radicals scavenging, immunostimulation, thermoregulation, protection from ultraviolet radiation, and have antiviral and antimicrobial properties [18,19,20]. Due to their antioxidant activity, melanins can be used to reduce metal compounds and thus synthesize metal nanoparticles. In contrast to traditional techniques for obtaining nanomaterials, the use of melanins can enable processes that are relatively fast and inexpensive, making them environmentally friendly [21,22]. Due to the aforementioned properties of melanins, they can carry out many active functions, which have resulted in numerous works describing the use of these pigments in combination with various polymers, including, although not limited to, bioactive food packaging [23].

Zinc oxide nanoparticles (ZnONP) are among the most commonly produced nanomaterials, next to titanium oxide and silicon dioxide NPs. As with other nanomaterials, the size and shape (and thus properties) of ZnONP depends on the preparation method. ZnONP, similar to titanium dioxide NP have a high ability to absorb ultraviolet radiation with a high band gap, making them transparent to visible light [24]. As a result, nano ZnO is commonly used in cosmetics, such as sunscreens, for protecting the skin against ultraviolet radiation. Additionally, ZnONP possess antimicrobial activity, making them suitable for the purification of water from microbial contaminants [25] or as antibacterial additives to paints and coatings [26,27]. Importantly, ZnONP are regarded as being safe (GRAS) by the United States Food and Drug Administration (USFDA, 21CFR182.8991). Combined, these properties make them very attractive for applications in active packaging systems [28].

Silver nanoparticles (AgNP) also possess potent antimicrobial properties, making them highly valued in the pharmaceutical and medical industries. As shown in numerous studies, AgNP are able to induce pore formation in bacterial cell membranes, most likely through the interaction of silver with sulfur present in membrane proteins. Further, when silver penetrates into the cell, it can interact with genetic material, causing its condensation inhibiting replication and gene expression [29,30,31,32]. Due to these properties, AgNP are used in various biomedical applications, such as in burn wound dressings and as catheter coatings. However, AgNP are also used in electronics, photonics, cosmetics, cleaning agents, and as disinfectants [26,33].

The antimicrobial and UV barrier properties of ZnONP and AgNP described above, along with potential other effects on the polymer matrix, provide prospective applications for these nanoparticles in active packaging materials. Nevertheless, the amount of nanoparticles loaded into the polymer matrix is crucial for the properties of the polymer, which as a consequence may deteriorate the quality of the packaging. As it has been shown for chitosan nanoparticles, the use of other than optimal amounts (smaller or larger) can affect the thermal, mechanical, barrier or optical properties [34]. 

In this work, our aim is to investigate the effect of the addition of melanin obtained from watermelon seeds, a food industry waste, on the properties of alginate films. The melanin was used as an additive directly or was used for synthesis of ZnONP and AgNP. To the best of our knowledge, there have been no reports on the modification of alginate films with either natural melanin or in combination with nanoparticles to improve the functionality of these materials. The chemical composition of the films after incorporation of melanin and nanoparticles was examined by FT-IR spectroscopy. In addition, the effect of melanin on the color, hydrodynamic and optical properties of the films was evaluated. Moreover, their potential bio(functionality) was assessed by evaluating their mechanical and barrier properties, as well as antioxidant and potential cytotoxicity in vitro.

## 2. Materials and Methods

### 2.1. Materials and Reagents 

Calcium chloride, hydrogen peroxide, disodium phosphate, monosodium phosphate, 1,1-diphenyl-2-(2,4,6-trinitrophenyl) hydrazyl (DPPH), 2,2’-azino-bis(3-ethylbenzothiazoline-6-acid) sulfonic acid (ABTS), potassium persulfate, potassium hexacyanoferrate (III), trichloroacetic acid, iron (III) chloride, iron (II) sulfate, tris (hydroxymethyl)aminomethane, pyrogallol, sodium alginate (M_w_ = 1,450,000), and *o*-phenanthroline were purchased from Sigma-Aldrich (Darmstad, Germany). Glycerol, ammonia water, hydrochloric acid, sodium hydroxide, chloroform, ethyl acetate, ethanol and methanol were from Chempur (Piekary Śląskie, Poland). Zinc (II) nitrate, peptone water and MacConkey medium were from Scharlau Chemie (Barcelona, Spain). Silver (III) nitrate was from POCh (Gliwice, Poland). Chapman’s medium was from Merck (Dramstadt, Germany). All reagents were of analytical grade.

The microorganisms used to evaluate the antimicrobial properties of the films were obtained from the American Type Culture Collection (ATCC). The strains used were *Escherichia coli* ATCC8739 and *Staphylococcus aureus* ATCC12600.

For in vitro cytotoxicity studies, murine fibroblast cell line (L929), as well as all cell culture reagents: Dulbecco’s Modified Eagle Medium (DMEM), fetal bovine serum (FBS), L-glutamine, penicillin, streptomycin, and all other cell culture reagents were purchased from Sigma-Aldrich (Poznań, Poland). All sterile, single-use cell culture plasticware was purchased from VWR (Gdańsk, Poland).

### 2.2. Preparation of Alginate Films

Fresh Crimson Sweet watermelons (*Citrullus lanatus*) were purchased from a local market (Szczecin, Poland). Melanin isolation and purification was performed as previously described [35]. Briefly, watermelon seeds (5 g) washed in distilled water and dried were immersed in 50 mL of 1 M NaOH for 24 h with shaking (150 rpm, 50 °C). The resulting mixture was then centrifuged (6000× *g* rpm, 10 min) and the separated supernatant was brought to pH 2.0 by adding 1 M HCl and followed by centrifugation (6000× *g* rpm, 10 min). The resulting precipitate was hydrolyzed with 6 M HCl (90 °C, 2 h), centrifuged again (6000× *g* rpm, 10 min) and washed five times with distilled water. Finally, the obtained precipitate was washed with chloroform, ethyl acetate and ethanol, dried and ground in a mortar. To prepare melanin containing films, distilled water (400 mL) was poured into 500 mL bottles and melanin was added in appropriate amounts (0.008 g, 0.02 g and 0.04 g) in order to obtain melanin concentrations of 0.10%, 0.25% and 0.50% (*w*/*w*), respectively. Ammonia water (2 mL) was added to all samples to create an alkaline environment that facilitated melanin dissolution. The bottles were placed on a shaker overnight to completely dissolve the melanin. The resulting solutions were pressure filtered to separate insoluble residues. The solution bottles were then placed on magnetic stirrers and 8 g of alginate was slowly added to each bottle. They were then placed back on the shaker overnight at 60 °C to completely dissolve the alginate. Glycerol, a plasticizing agent, was added (30% (*w*/*w*) relative to the amount of alginate used) to the solutions and stirred on a magnetic stirrer for 10 min. A control sample without melanin was prepared in the same fashion. To prepare films, alginate solutions were poured into square polystyrene plates (120 mm × 120 mm) at 40 g of solution per plate and dried at 40 °C for 48 h. All assays were performed in eight replicates.

Samples of AgNP or ZnONP were prepared using the same general procedure, however, with the addition of silver nitrate or zinc nitrate in an aqueous solution of melanin. For this purpose, aqueous solutions of silver nitrate or zinc nitrate were slowly added dropwise to the melanin solutions using a pipette until 10 mM was obtained in the film-forming solution. The resulting solutions were then incubated at 90 °C for 1 h. The solutions were cooled to room temperature before adding alginate (8 g). To prepare the films, the alginate solutions were poured onto square polystyrene plates (120 mm × 120 mm) at 40 g of solution per plate and dried at 40 °C for 48 h. All experiments were performed in eight replicates.

### 2.3. Characterization on Nanoparticles

NP purification was performed prior to performing the analyses. For this purpose, the mixtures were centrifuged (ZnONP mixtures at 5000 rpm and AgNP at 14,000 rpm) for 5 min and then the resulting precipitate was washed with distilled water. The procedure was repeated three times.

Samples of mixtures of melanin with AgNP or ZnONP were filtered using syringe filters (pore size 0.45 µm) and 1 mL of the obtained filtrates analyzed using UV-Vis light absorption spectroscopy (Thermo Scientific (Waltham, MA, USA) Evolution 220 UV-Vis spectrophotometer). The spectra were collected over the wavelength range of 300–800 nm, with a resolution of 1 nm. Melanin-nanoparticles mixture were also dried overnight at 40 °C and the resulting powder was collected for chemical composition analysis using a Perkin Elmer Spectrum 100 FT-IR spectrophotometer (Waltham, MA, USA). The obtained powders were measured directly using attenuated total reflection (ATR). The spectra were recorded over a wavelength range of 650–4000 cm^−1^, with a resolution of 1 cm^−1^. 

Dynamic light scattering (DLS) measurements were performed using Malvern Nanosizer ZS instrument (Worcestershire, UK) with a He-Ne laser source (633 nm). For each sample, three measurements were performed at each of two sample dilutions. For ZnONP, after purification, each pellet was re-dispersed in 2 mL of saline, yielding homogenous latté-colored suspensions. These suspensions were then further diluted 1:80 and 1:200 for measurements. For AgNP, after purification, pellets were dispersed in 1 mL of water, yielding pale dispersions, ranging from pink (0.5% MEL) to purple (0.1% MEL). These dispersions were measured neat, as well as after further 1:1 dilution with water. 

Each DLS measurement was performed at 25 °C after 2 min of temperature equilibration and consisted of 10–15 runs, at 173° scattering angle, and using automatic attenuation to ensure count rates <500 kcps. Data presented consist of hydrodynamic diameter (intensity-weighted average, “z-average” diameter) and polydispersity index (PDI) obtained from cumulant analysis using Malvern Zetasizer software v3.30.

### 2.4. Biocomposite Film Characterisation

#### 2.4.1. Determination of Moisture Content and Water Solubility

Moisture content (MC) was determined as the change in film (2 cm × 2 cm) weight after drying at 105 °C for 24 h. Water solubility (WS) was tested by adding pre-weighed film fragments (2 cm × 2 cm) to 30 mL of distilled water in conical tubes and stirring overnight. The samples were then centrifuged and the water was removed from the precipitate using a pipette. The samples were dried overnight at 60 °C and finally weighed again. Each material was tested in three replicates and mean values were calculated. The solubility of the films in water was calculated using the following formula:

(1) $$S\;\left(\%\right)=\frac{W_{1}-W_{2}}{W_{1}}\times 100\;$$

where *W*_1_ is the initial and *W*_2_ the final weight of the films.

#### 2.4.2. Thickness, Mechanical, and Thermal properties of Alginate Films

Film thickness was measured using an electronic thickness gauge (Dial Thickness Gauge 7301, Mitoyuto Corporation, Kangagawa, Japan) with an accuracy of 0.001 mm. Each sample was measured 10 times at randomly selected points and the results were averaged.

The tensile strength and elongation at break of the films were assessed using a Zwick/Roell 2.5 Z static testing machine (Ulm, Germany). The tensile clamp spacing was 25 mm and the head travel speed was 100 mm/min.

Differential scanning calorimetry (DSC) was used to assess thermal properties (DSC 3, Mettler-Toledo LLC, Columbus, OH, USA) using sequential heat-cool-heat cycles over a temperature range of 30–300 °C at *ϕ* = 10 °/min, under nitrogen flow (50 mL/min).

#### 2.4.3. Water Vapor Transmission Rate (WVTR) of Films

The water vapor permeability test (WVTR) was carried out using a gravimetric method: moisture sorption by pre-dried calcium chloride (9 g) was examined in containers tightly covered with test films (8.9 cm^2^). The containers were weighed daily over a period of three days (starting at day zero every 24 h until day three of the start of the analysis) to monitor the weight gain of calcium chloride and thus the water vapor permeability of the films. Each material was tested in four replicates and mean values for each day were calculated to express WVTR in g/(m^2^ × day) [36].

#### 2.4.4. Spectral Analysis of Films

UV-Vis spectra of films were measured using a Thermo Scientific (Waltham, MA, USA) Evolution 220 UV-vis spectrophotometer. Strips of films, with a surface area matching the quartz cuvette (5.5 cm × 1 cm), were placed along with a cuvette in the apparatus and spectra were recorded over a wavelength range of 300–800 nm, with a resolution of 1 nm.

The chemical composition of the obtained films was also evaluated using a Perkin Elmer Spectrum 100 FT-IR spectrophotometer (Waltham, MA, USA). Pieces of the films were measured directly, in ATR mode (32 scans per sample), and spectra were recorded over a wavelength range of 650–4000 cm^−1^, with a resolution of 1 cm^−1^.

#### 2.4.5. Film Color Analysis

The effect of melanin and silver or zinc nanoparticles on the color of the obtained films was assessed using a colorimeter (CR-5, Konica Minolta, Tokyo, Japan). Each sample was tested 10 times, at randomly selected points. The results (mean ± standard deviation) were expressed as L*, a* and b* parameters. In addition, Δ*E* (color difference) and *YI* (yellowing index) were calculated for comparison with unmodified alginate films, using the following equations:

(2) $$\Delta E={\left[{\left(L_{standard}-L_{sample}\right)}^{2}+{\left(a_{standard}-a_{sample}\right)}^{2}+\left(b_{standard}-b_{sample}\right)\right]}^{0.5}\;$$

(3) $$YI=142.86b\cdot L^{-1}\;$$

#### 2.4.6. Antioxidant Potential of Films

The antioxidant activity was measured by reducing power and free radical scavenging activity (ABTS^+^, DPPH, and superoxide ($O_{2}^{-}$) radicals) techniques as previously described [37], with minor modifications. For each film, two samples were tested and each measurement was performed in triplicate.

To assess reducing power, 100 mg film samples cut into pieces were placed in 1.25 mL of phosphate buffer (0.2 M, pH 6.6) and then 1.25 mL of 1% potassium ferricyanide solution was added. The samples were incubated at 50 °C for 20 min, after which 1.25 mL of trichloroacetic acid was added. The samples were then centrifuged at 1107 RCF for 10 min. From each sample, 1.25 mL of supernatant was taken, diluted with an equal volume of distilled water, and 0.25 mL of 0.1% ferric chloride solution was added. Finally, the absorbance of the samples was measured at 700 nm.

Free radical scavenging activity assay was performed against ABTS^+^, DPPH, and superoxide ($O_{2}^{-}$) radicals. The ABTS^+^ radical was induced prior to the addition of the film by mixing 5 mL of 7 mM ABTS with 2.45 mL of potassium persulfate and allowing it to stand overnight at room temperature, in the dark. ABTS^+^ solutions were then prepared by diluting with ethanol to an absorbance of 0.7. For each test, 100 mg of film was added to 10 mL of ABTS^+^ solution and incubated for six minutes in the dark. As negative control, polypropylene film with the same size as the samples was used, whereas the prepared ABTS^+^ solution without any film was used as a blank control. The mixtures were incubated under identical conditions. The absorbance of the samples was measured at 734 nm and the free radical scavenging activity was calculated using the following equation:

(4) $$Free\;radical\;scavenging\;activity\;\left(\%\right)=\frac{A_{sample}-A_{control}}{A_{sample}}\times 100$$

where *A_sample_* is the absorbance of the ABTS^+^ solution with the addition of the tested films, and *A_control_* is the absorbance of the blank ABTS^+^ solution.

For DPPH radical scavenging activity tests, 100 mg of each film was placed in 10 mL of 0.01 mM DPPH in methanol and incubated in the dark for 30 min. As negative control, polypropylene film with the same size as the sample was used, whereas the prepared DPPH solution without any film was used as a blank control. The mixtures were incubated under identical conditions. Absorbance was measured at 517 nm and free radical scavenging activity was calculated using the same equation as for ABTS.

Superoxide ($O_{2}^{-}$) radical scavenging activity was evaluated using the pyrogallol oxidation inhibition assay. For this purpose, 100 mg of each film was incubated for 5 min in 3 mL of 50 mmol/L Tris-HCl buffer (pH 8.2), with gentle stirring every minute. Then, 0.3 mL of pyrogallol was added. After exactly four minutes, the reaction was stopped by adding 1 mL of 10 mM HCl and the absorbance was immediately measured at 318 nm. As negative control polypropylene film was used. The radical scavenging activity was calculated using the following equation:

(5) $$O_{2}^{-}\;inhibition\;\left(\%\right)=\left[1-\frac{A_{1}-A_{1}^{’}}{A_{0}}\right]\times 100$$

where *A*_1_ is the absorbance of the reaction mixture with the addition of the sample, *A*’_1_ is the absorbance of the reaction mixture with water instead of pyrogallol and *A*_0_ is the absorbance of the reaction mixture without addition of test samples.

#### 2.4.7. Antimicrobial Activity 

The antimicrobial properties were investigated by measuring the inhibition of microbial growth on plate count agar (PCA) in the presence of discs made of test films. For *Escherichia coli*, MacConkey agar was used, while for *Staphylococcus aureus*, Chapman agar was used. After a day of culture, single colonies were picked from the media and added to sterile peptone water prepared according to the manufacturer’s instructions until an optical density of 0.5 McFarland was achieved. The microorganisms were then seeded onto PCA plates and two discs (2 cm in diameter) of each film were placed on top. After 24 h of culture, the zone of growth inhibition was measured.

#### 2.4.8. Evaluation of Cytotoxicity

Cytotoxicity screening was performed using a direct contact assay, as described previously [37]. L929 cells were maintained in culture using DMEM containing 10% FBS, 2 mM l-glutamine, 100 U/mL penicillin, and 100 µg/mL streptomycin. For each test film, discs (8 mm in diameter) were cut using a steel punch, followed by sterilization with 20-min UV exposure in a BSL-2 safety cabinet (Telstar Bio II Advance, Barcelona, Spain). 

For each experiment, L929 cells (passage 7–16) were trypsinized, counted using a hemocytometer, and seeded in the wells of 48-well plates (30,000 cells per well). After 24 h of incubation to permit the cells to adhere and spread, the media was aspirated and discs of each test film (5–6 samples each) were placed directly onto the cell monolayer—one well at a time. Manipulation of the thin discs was performed using a sterile 21 G needle: discs were “speared” vertically (from above), lifted, positioned in place in the well, and released by a gentle twist of the needle. Afterwards, 0.25 mL of fresh media was gently pipetted on top. As a sham control for normalization, wells with cells were aspirated, although no disc was placed, prior to adding 0.25 mL of fresh media. After a further 24 h of culture, cell viability was assessed by inverted light microscopy (Delta Optical IB-100, Minsk Mazowiecki, Poland) and using the resazurin assay [38]. Note that it was not possible to remove the discs, as they partially dissolved and in the case of samples containing nanoparticles, the nanoparticles interfered with the resazurin assay. Fluorescence plate reader measurements (Biotek Synergy HTX, Winooski, VT, USA, excitation 540 nm, emission 590 nm) were converted to normalized cell viability as percentage of sham by first subtracting the mean signal of six blank wells (no cells) from all values, followed by dividing by the mean signal of the sham wells (n = 6).

### 2.5. Statistical Analyses

All analyses were made at least in triplicate. Statistical analysis was performed using Statistica version 13 software (StatSoft Poland, Krakow, Poland). Differences between means were determined by analysis of variance (ANOVA) followed by Fisher’s post hoc LSD test at a significance threshold of *p* < 0.05.

## 3. Results and Discussion

### 3.1. Characterization of Nanoparticles 

**Figure 1:**
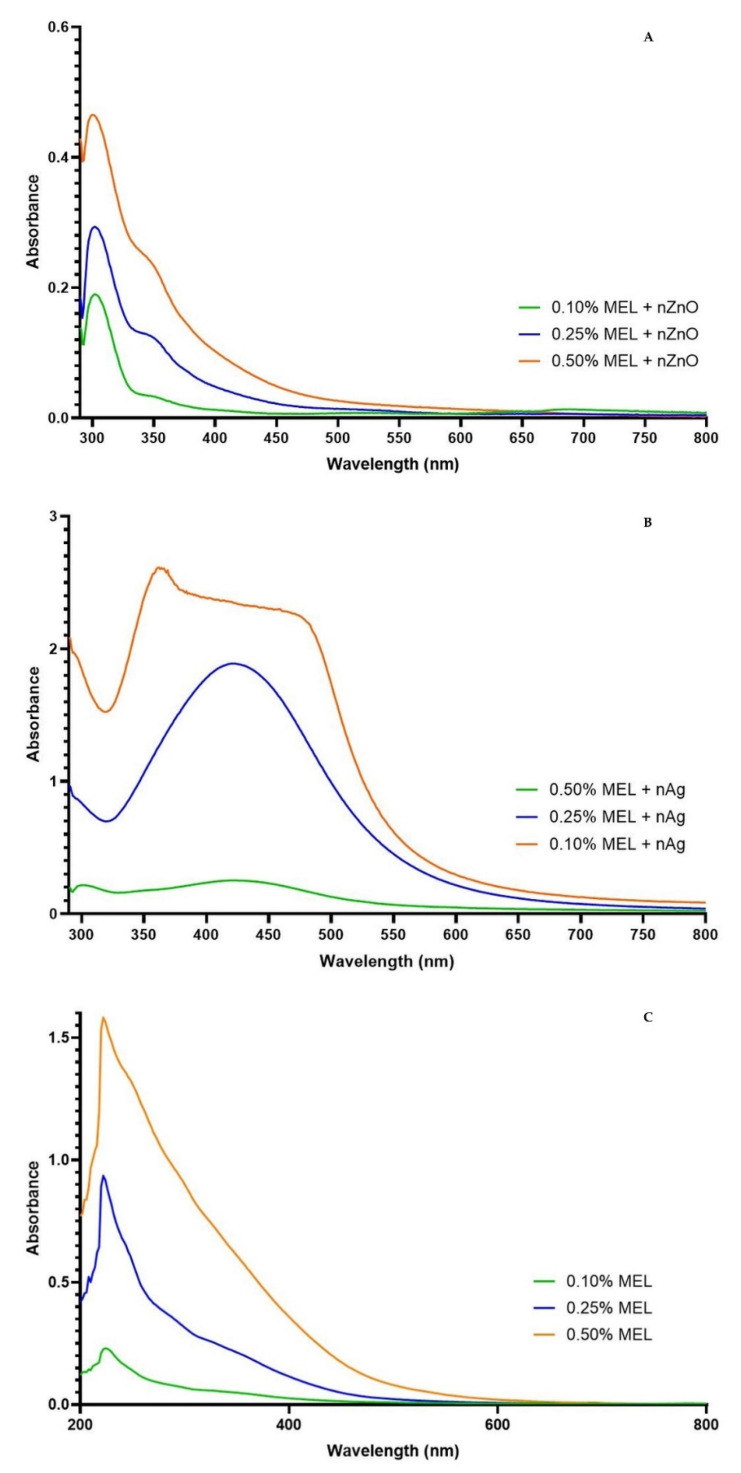
UV-Vis spectra of obtained zinc oxide nanoparticles (**A**), silver nanoparticles (**B**) samples, UV-Vis spectra of *C. lanatus* melanin at different concentrations for comparison (**C**).

In order to investigate the quality of obtained nanoparticles, UV-Vis analysis was carried out. UV-Vis spectra of ZnONP are shown in Figure 1A. The absorption maximum was at 307 nm. This value is shifted towards the UV-B radiation, as compared to the results obtained by Roy and Rhim [22], who noted a maximum at 365 nm for ZnO nanoparticles prepared using melanin.

UV-Vis spectra of AgNP are shown in Figure 1B. The maximum absorption peak can be observed at 420, 421, and 361 nm for 0.10% MEL + nAg, 0.25% MEL 0.25% + nAg, and 0.50% MEL + nAg, respectively. These results are similar to data obtained by Shankar and Rhim [39], as well as Vilmala et al. [40].

**Figure 2:**
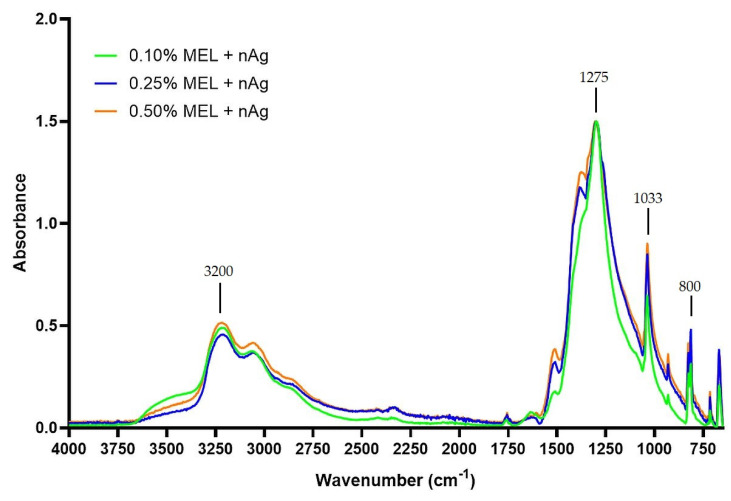
FTIR spectra of obtained silver nanoparticles samples.

In order to investigate the presence of characteristic bonds in the samples FTIR analysis was performed. Both silver and ZnONP samples may contain melanin used in the synthesis of these nanoparticles, which was assessed using FTIR. Figure 2 shows the FTIR spectra of the AgNP obtained from the 0.50% MEL + nAg sample. Several distinct peaks can be seen. The stretching vibration of –OH bonds (phenols) at 3200 cm^−1^ are likely from the melanin present in the sample, along with the peaks at 1275 cm^−1^ and 1033 cm^−1^ corresponding to C–O stretching, and 800 cm^−1^, which can be attributed to aromatic groups. These results are similar to measurements of AgNP biosynthesized using plant extracts [41].

**Figure 3:**
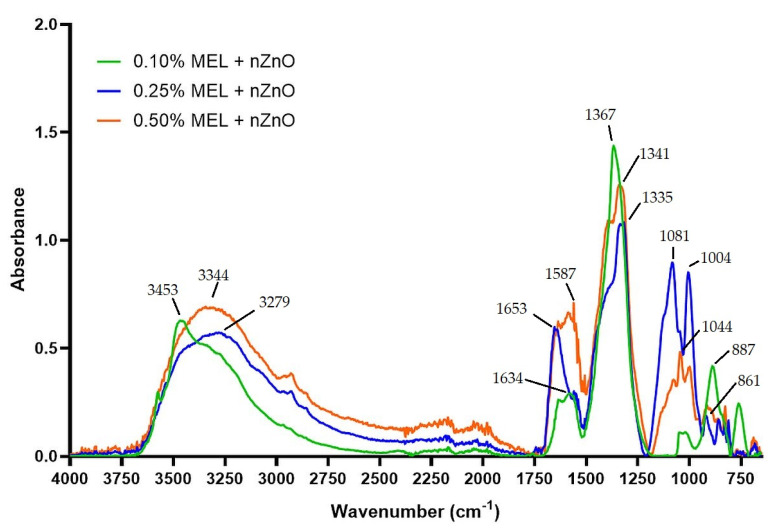
FTIR spectra of obtained zinc oxide nanoparticles samples.

Figure 3 shows the FTIR spectra of ZnONP. Considerable differences in the intensity and position of the FTIR peaks can be noted for the individual samples. This is likely due to the different amounts of residual melanin due to the different concentrations used during preparation. The primary peaks occur in the spectral regions: 3500–3250 cm^−1^, 1600–1550 cm^−1^, 1400–1300 cm^−1^, 1100–950 cm^−1^, and 900–750 cm^−1^. These results are in good agreement with data obtained by Roy and Rhim [22], who also used melanin for ZnONP synthesis.

In order to investigate average particle size, dynamic light scattering was used. Dynamic light scattering results of the obtained particles are presented in Table 1. For all ZnONP samples, unimodal, yet broad distributions were obtained, with a trend towards smaller particles with higher MEL content. At 0.50% MEL, the particles were the smallest, sub-1-micron in diameter and with the narrowest distribution (PDI = 0.266 ± 0.061). For the case of AgNP, particle sizes were markedly smaller, all <300 nm, and the effect of MEL concentration was different: the highest MEL concentration (0.50%) yielded the largest particles, although again with the narrowest distribution (PDI = 0.288 ± 0.037). Additionally, for the 0.10% MEL AgNP, a minor shoulder was observed (~10% of area) at ~60 nm diameter.

### 3.2. Hydrodynamic Properties (Moisture Content and Water Solubility) and Water Vapor Transmission Rate (WVTR)

In order to investigate the influence on hydrodynamic properties of the addition of melanin, ZnONP and AgNP, moisture content, water solubility and water vapor transmission rate have been examined. The moisture content and water solubility of the unmodified and modified alginate films are shown in Table 2. The moisture content of the unmodified alginate film was 11.61 ± 0.22%. The addition of melanin caused a significant increase to 14.93 ± 3.14% for ALG + 0.10% MEL film (*p* < 0.05). These values are similar to those reported for agar films with melanin [42]. While the effect of melanin addition on the moisture content of the films was consistent with the results of other researchers [22,37,43,44], there are also reports of the opposite effect of melanin [45,46]. These differences can be attributed to the different properties of the biopolymers used and the resulting different interactions between their functional groups with melanin, leading to an increase or decrease in the overall availability of free hydroxyl groups able to bind water molecules [46]. 

In contrast to melanin, the addition of AgNP caused a decrease in MC, to a range from 10.96 ± 1.42 to 11.61 ± 0.29%, which is comparable to the results of Rhim et al. [47]. However, the addition of ZnONP to the films did not affect the MC of the obtained films. Interestingly, Kotharangannagari and Krishnan [48] observed a decrease in moisture content with increasing amount of zinc oxide added to films made of starch with lysine and various concentrations of ZnONP. This again indicates that the specific interactions between the biopolymer and the nanoparticle drive film affinity for moisture.

The water vapor barrier properties of the tested films are presented in Table 2. Compared to control alginate films, the WVTR of all of the modified alginate films had lower WVTR, with the effect being more pronounced with increasing concentration in the melanin used. For MEL addition alone, the WVTR drops from 1589.70 ± 54.96 g/(m^2^ × Day) for neat alginate films to 1117.23 ± 285.85 g/(m^2^ × Day) for the highest melanin concentration. As reported by Bang et al. [49], the effects of melanin on water vapor barrier properties can be attributed to the interactions of melanin with the polymer matrix, especially with free hydrophilic chains. As a result, previous studies have found both positive effects of melanin on WVTR [49], as well as negative effects [36,37] and a variable effect dependent on the melanin concentration [44]. 

Compared to the addition of melanin, the presence ZnONP and AgNP resulted in a smaller change in WVTR through the films. At the highest concentration of melanin, the WVTR was 1463.20 ± 116.17 and 1229.34 ± 57.05 g/(m^2^ × Day), for ZnONP and AgNP, respectively. However, both of these values remain lower than control alginate. For the case of ZnONP, the reduced effect may be attributed to their much larger size (~1 µm). In previous work, Roy and Rhim [22] studied carrageenan films with the addition of ZnONP synthesized with melanin, similar to this work. They observed that the addition of ZnONP nanoparticles increased the barrier properties of the film. Likewise, Kanmani and Rhim [50] as well as Shankar et al. [51] obtained similar results. However, for PLA, Chu et al. [52] observed an increase in water permeability through films after adding ZnONP, although no significant effect of the addition of AgNP. Further, Shankar and Rhim [39] noted a slight increase in water vapor permeability after adding AgNP to agar films, whereas Rhim et al. [53], in a similar test system, noted a decrease in this parameter with increasing silver nanoparticle concentration. Overall, the water vapor barrier properties are highly dependent on the properties of the materials involved, their interactions, and the preparation, and dispersion of the nano-additives used.

In terms of water solubility, the addition of melanin reduced solubility from 79.70 ± 11.26% to 73.54 ± 14.05% for ALG + 0.10% MEL and 75.68 ± 9.64% for ALG + 0.50% MEL, although the differences were not significant (*p* > 0.05). A similar effect of melanin was previously described for whey protein isolate and concentrate films, as well as for gelatin films [33,47]. However, Roy et al. [43] noted an inverse relationship for chitosan films, attributed to interactions of melanin with polymer chains. 

Meanwhile, films with AgNP had lower solubility (61.92 ± 1.68% for ALG + 0.10% MEL + nAg) than the controls, however, as the amount of melanin in the film increased, the solubility significantly increased, to 69.79 ± 2.19% (*p* < 0.05). The same relationship was also observed for the samples with ZnONP: a trend of increasing water solubility from 69.37 ± 3.86% to 73.23 ± 2.50% with increasing melanin concentration. Previously, Rhim et al. [45] reported an increase in the solubility of chitosan films after the addition of nanosilver, although the differences were not statistically significant.

### 3.3. The Thickness, Mechanical and Thermal Properties

The thickness, mechanical and thermal properties of the tested films are summarized in Table 3. In general, the addition of melanin did not have a significant effect on the thickness of the films. This is in good agreement with prior work with films made of whey protein concentrate/isolate [37], although not with those made of agar [42], gelatin [54], carrageenan [22] or polybutylene adipate terephthalate (PBAT) [49] where melanin addition tended to increase film thickness. Meanwhile, for carboxymethyl cellulose (CMC) films, melanin had no effect on the film thickness [36]. 

Regarding mechanical properties of the films, the tensile strength (TS) increased for films with increasing melanin content from 5.97 ± 1.12 MPa for films with 0.10% (*w*/*w*) melanin content to 17.59 ± 3.91 MPa for films with the highest content. An upward trend was also be observed for the films with the addition of nanoparticles, although the effect was not as pronounced. The results are broadly similar, however lower in magnitude than those obtained by other authors [22,36,42,44,45,49,54,55]. Elongation at break (EB) also increased with increasing melanin content. However, previous studies suggest that the value of the elongation at break may increase with increasing melanin concentration up to a point, beyond which it begins to decline; the same relationship may also exist for the tensile strength [43,49,55].

Surprisingly, the addition of ZnONP decreased the values of both tensile strength (TS) and elongation at break (EB) to 6.10 ± 3.06% and 1.60 ± 0.32%, respectively. Previously, Roy and Rhim [22] had found no significant changes for carrageenan films after the addition of these nanoparticles. For non-biopolymers the literature is mixed: Mania et al. [56] reported an increase in both of these parameters for polyethylene films due to the action of nano ZnO, whereas Chu et al. [52] recorded a decrease in TS and an increase in EB for poly (lactic acid) (PLA) films.

In contrast to ZnONP, AgNP caused an increase in tensile strength to approximately 15 MPa and a decrease in elongation at break to about 2% for all samples. In contrast, Rhim et al. [53], did not observe significant changes in EB after adding these nanoparticles to agar films, however, TS values were lower at low concentrations of nanoparticles used and then increased with the increase in the amount of AgNP. However, Shankar and Rhim [39] showed a decrease in TS for agar films, with a simultaneous increase in EB value. Again, the literature is mixed for non-biopolymer PLA: Chu et al. [52] observed that TS decreased slightly and EB slightly increased, while Fortunati et al. [57] found that both parameters tended to decrease with AgNP addition.

Overall, the results obtained for alginate films and the results reported in the literature for various polymer matrices indicate that the addition of both melanin and silver and ZnONP can have different effects, i.e., positive, negative or no change in the described mechanical parameters, depending on the type of polymer used, the amount of melanin/nanoparticles used, as well as the methods and conditions of nanocomposites preparation.

Table 3 presents the melting temperatures (T_m_) and melting enthalpies (∆H_m_) of the films as measured by DSC. Compared to the neat alginate films, the addition of melanin decreased the T_m_ for films containing the highest concentration of melanin (from 127.5 ± 1.9 °C to 99.8 ± 1.5 °C), with ∆H_m_ also decreasing with increasing melanin concentration (from 106.0 ± 13.6 J/g to 66.7 ± 8.5 J/g). For both ZnONP and AgNP, T_m_ decreased, while ∆H_m_ increased, although the values of these parameters remained similar for individual trials. This is in contrast to previous work with WPC/WPI films [37], where modification with melanin increased their thermal stability. However, addition of melanin to agar [42], cellulose [55] or PLA [44] films did not affect these parameters.

### 3.4. UV Barrier Properties

**Figure 4:**
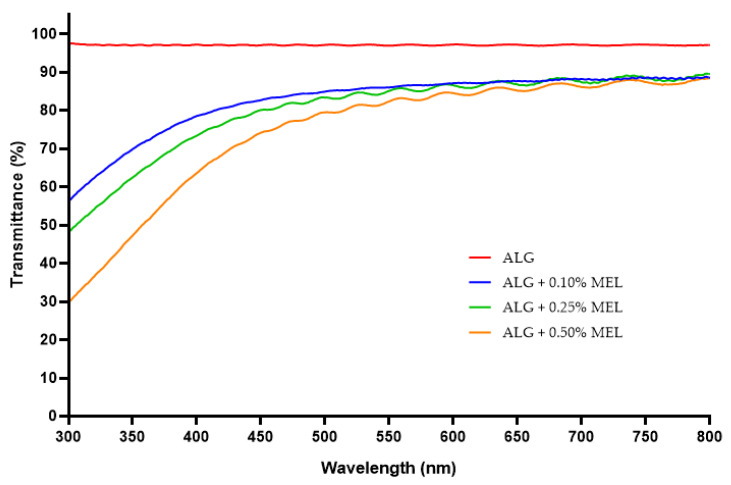
UV-Vis spectra of unmodified and melanin-modified alginate films.

To evaluate the UV-Vis barrier properties, spectrophotometric spectra of the obtained films were analyzed. Figure 4 presents the UV-Vis spectra of the unmodified alginate film and the alginate films modified with increasing concentrations of melanin. The unmodified alginate film exhibited very poor (almost no) barrier properties against visible wavelengths, UVA, and part of the UVB range (above 300 nm). The addition of melanin caused a similar decrease in the transparency for visible light, regardless of melanin concentration. However, in the range of blue light, UVA, and UVB, the barrier properties increased with increasing amounts of melanin. Even the lowest melanin concentration (0.10%) had a significant effect on the transmittance of light at specific wavelengths. As mentioned earlier, melanins are known to have very good light absorption in the UV range. As a result, similar effects of melanins on light transmission have been reported for films of gelatin [54], agar [42], carrageenan [43], PLA [44], cellulose [55], carboxymethylcellulose [36], PBAT [49], and chitosan [45].

**Figure 5:**
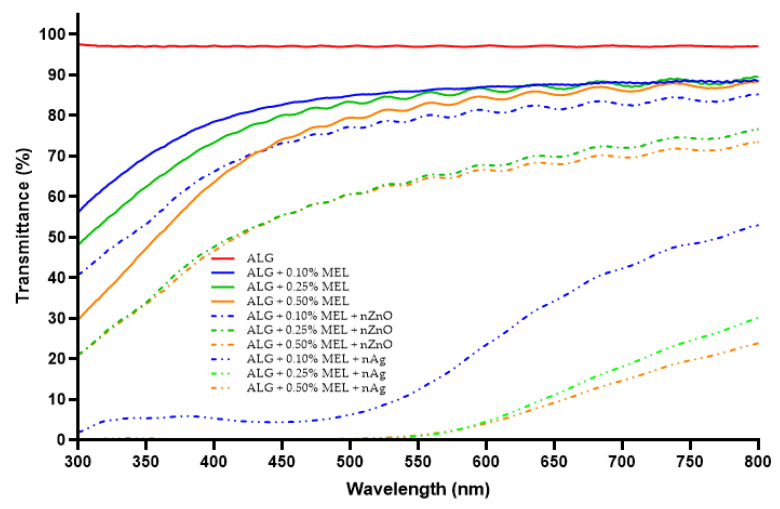
UV-Vis spectra of melanin-modified alginate film without or with the addition of zinc oxide silver nanoparticles.

Figure 5 presents a comparison of UV-Vis spectra of melanin-modified alginate films without and with the addition of ZnONP or AgNP. The ZnONP reduced the transmittance of the films by few to several percent in the visible and UV range. Metal oxide nanoparticles are commonly used in sunscreens due to their UV blocking properties [26,27]. Rashimi et al. [58] also observed an increased absorbance of UV-Vis radiation for polyvinyl alcohol films with the addition of ZnONP. A very significant effect can be observed with melanin concentrations of 0.25% and 0.50% resulting in essentially complete blocking of radiation with a wavelength shorter than 525 nm. For the sample with 0.10% melanin concentration, this parameter was also very low, on the order of 5 ± 0.5% transmittance. Very similar results were obtained by other researchers for films modified with AgNP, such as agar and chitosan films [39,40,53].

### 3.5. FT-IR Analysis

**Figure 6:**
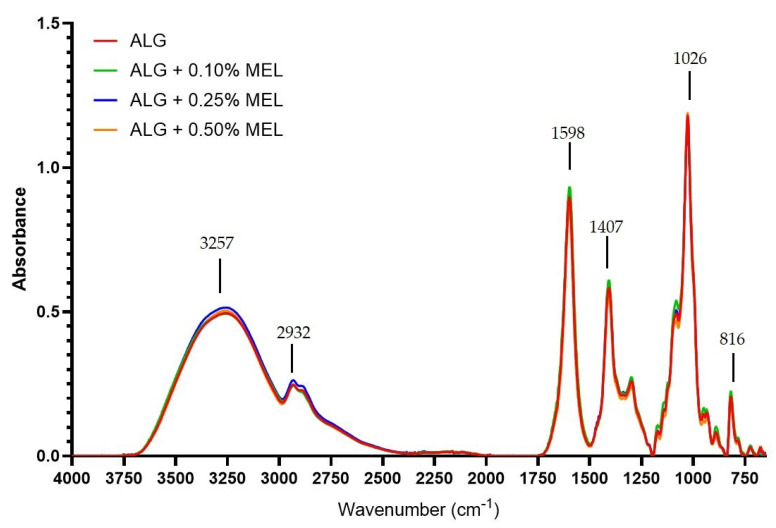
FT-IR spectra of unmodified and modified with melanin alginate films.

In order to investigate the presence of characteristic bonds in the samples FT-IR assay was performed. Figure 6 summarizes the FT-IR spectra of unmodified alginate film and melanin-modified alginate films without nanoparticles. The graph shows numerous bands in the wavenumber range between 600 cm^−1^ and 1750 cm^−1^ and bands in the range of 3700–3000 cm^−1^ corresponding to the stretching vibrations of the –OH bonds [59], and between 3000 and 2850 cm^−1^ caused by –CH stretching vibration [60]. The bands present in the range of 1750–600 cm^−1^ are characteristic of alginate and they are as follows: at 1598 cm^−1^ it is the result of asymmetric vibrations stretching the –CO bond in the –COO– group [59,60], at 1407 cm^−1^ caused by symmetrical stretching vibrations –CO in the –COO– group [61], at 1026 cm^−1^ associated with asymmetric vibrations –COC [60] and at 816 cm^−1^ being characteristic of the presence of mannuronic acid residues [61].

The addition of melanin did not affect the formation of new bonds or the disappearance of existing ones. The slight shifts in bands and changes in their intensity are probably due to interactions between alginate and melanin in the form of hydrogen bonds and van der Waals forces, which is consistent with the observations of other researchers [36,37,42,54].

**Figure 7:**
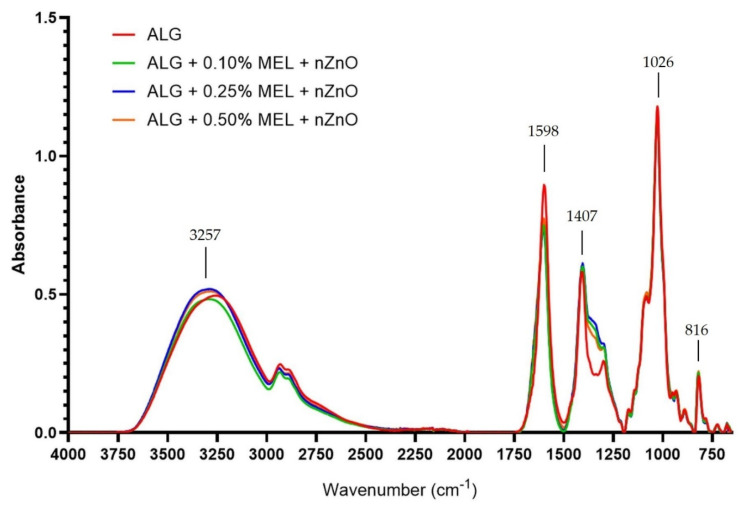
FT-IR spectra of unmodified and modified with melanin and zinc oxide nanoparticles alginate films.

The addition of ZnONP (Figure 7) weakened the band at 1598 cm^−1^ (asymmetric vibrations stretching the –CO bond in the –COO– group) and increased the intensity of the band at 1355 cm^−1^ (asymmetric bond vibrations –CH– [52]. For the remaining bands, the differences in the measured intensity were small. Similar observations were noted for films made with the use of carrageenan [22] and PLA [52], while in the case of film made of polyvinyl alcohol, researchers reported a weakening of the peak intensity with an increase in the amount of added ZnONP [58].

**Figure 8:**
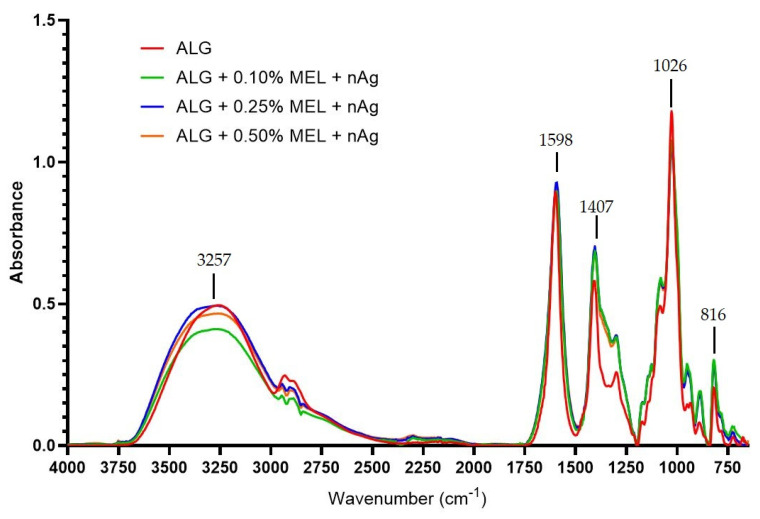
FT-IR spectra of unmodified and modified with melanin and silver nanoparticles alginate films.

In the case of AgNP (Figure 8), the intensity of the bands in the 1400–600 cm^−1^ range was enhanced. No additional peaks are visible, which indicates that the AgNP had no effect on the structure of the obtained alginate film. Shankar and Rhim [39], modifying the agar film with AgNP, also did not notice any changes in the chemical structure of the obtained product and their results show an increase in the intensity of most of the bands. However, to make their films, they used a concentration of silver nitrate that was twenty times lower, so the final content of nanoparticles was significantly lower. Vilmala et al. [40], on the other hand, noted a more pronounced effect of AgNP on films made of chitosan: AgNP addition caused not only a change in the intensity of some bands and their shift; it also resulted in the disappearance of some of bands and the appearance of new ones.

### 3.6. Color

**Figure 9:**
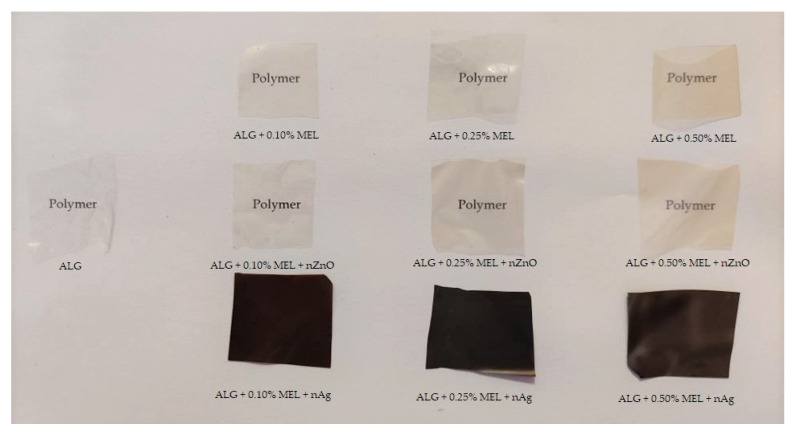
The visual appearance of neat and modified alginate films.

In order to investigate the influence of melanin and ZnONP and AgNP on color, chromatic parameter analyses were performed. Table 4 lists the chromatic parameters, the total color difference and the yellowness index of the unmodified alginate film and the film modified with melanin and with the addition of AgNP or zinc oxide, whereas Figure 9 shows the apparent color. As the melanin content increased, the parameter L * decreases slightly (the films become darker), while the parameters a * and b * increase (the red and yellow components of the overall color tone increase, respectively (*p* < 0.05)). The yellowness index also increases as the amount of melanin used increases (*p* < 0.05). The total color difference, for films containing only melanin, is noticeably different for each sample and is greater than unity, which is considered to be a difference noticeable to the human eye [44]. Similar relationships have been described in the literature for the effect of melanin addition on films made of gelatin [54], agar [42], carrageenan [43], chitosan [45], PLA [44], WPI/WPC [37], carboxymethylcellulose [36], and PBAT [49].

For the case of ZnONP, comparing the values of individual parameters for films with the same melanin content, both with or without nanoparticles, analogous changes can be observed, i.e., a decrease in the L* parameter and an increase in the other parameters. Roy and Rhim [22] obtained similar relationships for carrageenan film modified with ZnONP.

However, when making similar comparisons for films with and without AgNP, it can be seen that the L* parameter is over three-fold lower than for other types of films, which results from its much darker color. The parameters a* and b* have increased significantly, which indicates an increase in the red and yellow coordinates in the overall color tone of the film. This is due to the dark brown color imparted by the addition of nanosilver. The total color difference for these films has similar values, while the yellowness index is ten times higher than the results obtained for films with melanin alone. A similar effect of the addition of AgNP was described for agar film [39].

UV-barrier property (T_280_) and transparency (T_660_) of neat and modified films represented as transmittance at 280 nm and 660 nm, respectively, are shown in Table 4. Unmodified alginate films was transparent for both UV (96.95%) and visible (97.01%) light. As expected, based on melanin properties and visual appearance of the films, melanin-modified films were less transparent for both visual and UV light. UV light barrier properties increased as the amount of melanin used increased (from T_280_ equal to 61.22% for ALG + 0.10% MEL film to 43.67% for ALG + 0.50% MEL). ZnONP addition effected a further increase in visual light barrier properties and slight increase in UV light barrier properties. Addition of AgNP resulted in nearly no transmittance for both UV and visible light, especially for ALG + 0.50% MEL + nAg sample (4.19% and 10.29%, respectively). Those results are in line with findings for alginate films modified with copper sulfide nanoparticles [62]

### 3.7. Antioxidant Activity

In order to investigate the antioxidant properties of the obtained films reducing power (RP) and the free radicals scavenging ability were studied. Table 5 presents the antioxidant properties of the films (RP), and the free radicals scavenging ability (DPPH, ABTS, $O_{2}^{-}$). As expected, the addition of melanin improved the properties of free radicals scavenging activity by the tested films from 8.60 ± 0.00% for DPPH and 7.27 ± 2.39% for ABTS to 90.62 ± 0.00% and 90.87 ± 0.02%, respectively, for the lowest melanin concentration used. Due to its insolubility in water and high antioxidant activity, all melanin-modified films exhibited ~90% DPPH and ABTS radicals scavenging regardless of the melanin concentration. For the reducing power, an upward trend was obtained with an increase in melanin concentration, while the ability to scavenge free radicals remains similar for all MEL concentrations. These results are consistent with previous studies that have shown that melanin has a significant effect on the scavenging of these radicals by various modified films [36,37,42,43,44,45,54,55].

The addition of ZnONP caused a decrease in RP and DPPH values to 0.016 ± 0.002 and 5.15 ± 0.00%, respectively, for the 0.10% melanin concentration. These values were lower than those for unmodified alginate films. Meanwhile, the ABTS and superoxide scavenging results were noticeably lower than for the film with melanin alone, however still several times higher than for the control alginate. For AgNP, on the other hand, the reduction power markedly increased. However, radical scavenging ability was the lowest of all modified films. To the best of our knowledge, there are few if any similar studies on the synergic effect of melanin and metal nanoparticles on the antioxidant properties of films based on polymer matrices. Therefore, further studies should be conducted to determine the relationship between these additives and the antioxidant activity of different polymer matrix films.

### 3.8. Antimicrobial Activity 

In order to test the antimicrobial properties of the obtained films, the ability to inhibit the growth of selected microorganisms was examined. Neither the unmodified alginate film nor the alginate films with the addition of melanin at various concentrations resulted in the formation of inhibition zones in any of the two tested microorganism strains. Previously, the lack of antimicrobial effect of melanin from the common mushroom (*Agaricus bisporus*) against the microorganisms used here was reported for PLA films [44]; however, the authors did note that melanin-modified materials were effective against *Enterococcus faecalis, Pseudomonas aeruginosa* and *P. putida*. On the other hand, melanin from *A. bisporus* did result in antimicrobial properties against *E. coli, S. aureus* and *Candida albicans* for the carboxymethylcellulose films. This again indicates that in this case the interactions of melanin with the polymer matrix have a significant influence on the properties of modified films.

For the films containing nanoparticles of silver or zinc oxide, the formation of zones of growth inhibition around the discs was observed. As illustrated in the pictures (supplementary materials), the nanoparticles migrate from the films into the medium. This effect was observed for all of the metal nanoparticle modified films tested. The sizes of the zones of growth inhibition for the tested microorganisms are presented in Table 6 and representative photos are shown in Supplementary Materials. The effects were similar for both types of nanoparticles against both microorganisms. The inhibitory effect of AgNP on the growth of *E. coli, Bacillus* and *Klebsiella pneumoniae* has been demonstrated for chitosan films [40], *E. coli* and *S. aureus* for PLA films [52,57], *E. coli* and *L. monocytogenes for* agar agar [39,53]. While chitosan film with the addition of cellulose dialdehyde nanocrystals showed inhibition for various strains of Gram positive, Gram negative bacteria and fungi [30]. Likewise, polymer films enriched with ZnONP have an antimicrobial effect well described in the literature; films with the addition of this nanomaterial showed antimicrobial activity for *E. coli* [22,52,56], *S. aureus* [56] and *L. monocytogenes* [22].

The genotoxic effects of ZnONP are based on the following mechanisms: generation of reactive oxygen species (ROS) in cells; attachment directly to DNA or during cell division; influence on chromosome disorders. These phenomena occur even at ZnONP concentrations of several μg/mL [63]. AgNP also exhibit genotoxic effects, among others by binding to genetic material and thus affecting its replication and gene expression, however the smaller the nanoparticles are, the more toxic they are [64]. 

### 3.9. Cytotoxicity Screening

**Figure 10:**
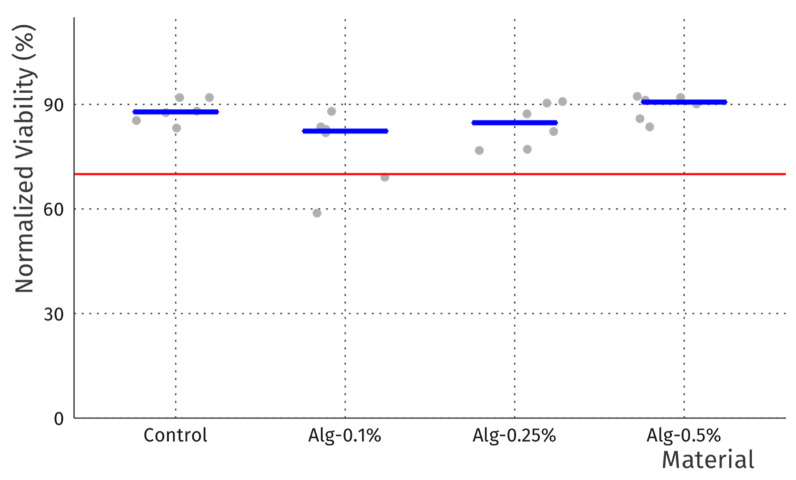
Cell viability data normalized to sham control (no disc) for alginate films with MEL and control film without MEL. Grey dots represent individual samples (n = 6), while blue bars indicate median values. Red line marks the 70% viability threshold of ISO10993-5.

In order to screen for potential cytotoxicity of the prepared films, we performed direct contact assays where discs of materials were placed directly on top of pre-seeded fibroblast cells (L929 cell line). As the samples partially dissolved it was not possible to remove them prior to microscopic observation or viability assay. However, for the case of alginate films with different amounts of MEL, no interference was observed and after 24-h incubation, no cytotoxicity was detected by resazurin viability assay (Figure 10) or microscopy (see Supplemental Information for representative micrographs). Viability was well in excess of the 70% threshold guideline of ISO10993-5 and no marked changes in morphology were observed. The experiment was repeated with similar results. These results are in good agreement with our previous work with similar films obtained from whey protein concentrate/isolate [37]. The minor ~10% reduction in viability compared to sham may be due to some mechanical damage to the monolayer while discs are placed, as well as possible diffusional limitations, which should be minor due to swelling and dissolution of the discs.

For the case of alginate films with ZnONP and AgNP, the presence of particles interfered with the resazurin viability assay and significantly impeded microscopy, as compared to the alginate films without particles—particularly for the case of AgNP. This is likely due to the differences in reducing power as well as optical properties of the films noted previously. Unfortunately, due to swelling and partial dissolution of the films during the 24 h incubation, it was not possible to remove the samples to improve assay and imaging conditions. However, despite significantly reduced contrast and resolution, we were able to confirm robust cell growth using inverted light microscopy, similar to that for cells exposed to films without nanoparticles (see Supplemental Information for representative micrographs). The experiment was repeated with similar results. As a result, we conclude that the addition of ZnONP or AgNP did not have an adverse effect on cell viability, which is consistent with the fact that these materials are commonly used in both the pharmaceutical and cosmetics industries. However, further studies may be needed to rule out any negative effects.

## 4. Conclusions

This paper studied the properties of alginate films modified with melanin obtained from watermelon seeds as a by-product of agricultural industry and with Ag and ZnO NP. The properties of melanin-modified films and films with NP were compared to unmodified alginate films and to each other. Melanin modification had a clear effect on mechanical, antioxidant (~90% ABTS and DPPH radicals scavenging for all melanin modified films), hydrodynamic and barrier properties. The nanoparticles exhibited synergistic (silver nanoparticles addition effected two-fold higher tensile strength or both nanoparticles effected in increase in UV-Vis barrier properties) or antagonistic effects (decrease in some antioxidant properties compared to melanin-modified films) on the described melanin properties and they have developed an antimicrobial effect (up to four mm grow inhibition zones of *E. coli* and *S. aureus* for both zinc oxide and silver NP). It can be concluded that the obtained biofunctional melanin-modified alginate films, due to their antimicrobial and antioxidant activities, may find application for packaging of active food or potentially for biomedical applications.

## Figures and Tables

**Table 1:** Dynamic light scattering results: hydrodynamic diameter (z-avg) and polydispersity index (PDI). Data are expressed as mean ± standard deviation (SD) of six measurements, 10–15 runs each.

Sample	Hydrodynamic Diameter (nm)	PDI
0.10% MEL + nZnO	1523 ± 201 ^a^	0.476 ± 0.172 ^a^
0.25% MEL + nZnO	1167 ± 74 ^b^	0.312 ± 0.104 ^a^
0.50% MEL + nZnO	988 ± 42 ^b^	0.266 ± 0.061 ^a^
0.10% MEL + nAg	230 ± 6 ^c^	0.412 ± 0.023 ^a^
0.25% MEL + nAg	216 ± 6 ^c^	0.359 ± 0.037 ^a^
0.50% MEL + nAg	265 ± 16 ^c^	0.288 ± 0.037 ^a^

Values are means ± standard deviation. Means with different letters (a–c) are significantly different at *p* < 0.05 (compared to other variants within groups).

**Table 2:** Moisture content (MC), water solubility (WS) and Water Vapor Transmission Ratio (WVTR) of unmodified alginate films and films modified with melanin and melanin with nanoparticles.

Sample	MC (%)	WS (%)	WVTR (g/(m^2^ × Day))
ALG	11.61 ± 0.22 ^a^	79.70 ± 11.26 ^a^	1589.70 ± 54.96 ^a^
ALG + 0.10% MEL	14.93 ± 3.14 ^bA^	73.54 ± 14.05 ^bA^	1240.92 ± 145.23 ^bA^
ALG + 0.25% MEL	12.20 ± 1.27 ^aB^	73.65 ± 5.93 ^bA^	1160.92 ± 284.22 ^bA^
ALG + 0.50% MEL	11.34 ± 0.31 ^aB^	75.68 ± 9.64 ^bA^	1117.23 ± 285.85 ^bA^
ALG + 0.10% MEL + nZnO	13.20 ± 0.24 ^aA^	69.37 ± 3.86 ^bA^	1549.44 ± 129.52 ^aA^
ALG + 0.25% MEL + nZnO	12.78 ± 0.62 ^aA^	72.28 ± 1.52 ^bA^	1503.18 ± 92.62 ^aA^
ALG + 0.50% MEL + nZnO	12.64 ± 0.40 ^aA^	73.23 ± 2.50 ^bA^	1463.20 ± 116.17 ^aA^
ALG + 0.10% MEL + nAg	10.96 ± 1.42 ^aA^	61.92 ± 1.68 ^bA^	1393.13 ± 89.11 ^bA^
ALG + 0.25% MEL + nAg	10.97 ± 0.03 ^aA^	66.66 ± 3.97 ^bA^	1341.85 ± 173.89 ^bA^
ALG + 0.50% MEL + nAg	11.61 ± 0.29 ^aA^	69.79 ± 2.19 ^bA^	1229.34 ± 57.05 ^bA^

Values are means ± standard deviation. Means with different lowercase (a–c) are significantly different at *p* < 0.05 (compared to control), means with different uppercase (A–B) are significantly different at *p* < 0.05 (compared to other variants within groups).

**Table 3:** Thickness, mechanical, and thermal characteristics of unmodified and modified alginate films.

Sample	Thickness (mm)	TS (MPa)	EB (%)	T_m_ (°C)	∆H_m_ (J/g)
ALG	0.041 ± 0.020 ^a^	7.59 ± 2.69 ^a^	2.16 ± 0.96 ^a^	127.5 ± 1.9 ^a^	103.0 ± 13.2 ^a^
ALG + 0.10% MEL	0.045 ± 0.028 ^aA^	5.97 ± 1.12 ^aA^	2.12 ± 1.12 ^aA^	128.1 ± 1.9 ^aA^	106.0 ± 13.6 ^aA^
ALG + 0.25% MEL	0.036 ± 0.020 ^aA^	7.26 ± 6.80 ^aA^	3.44 ± 1.97 ^bB^	128.0 ± 1.9 ^aA^	73.5 ± 9.4 ^bB^
ALG + 0.50% MEL	0.036 ± 0.014 ^aA^	17.59 ± 3.91 ^bB^	5.21 ± 1.40 ^cC^	99.8 ± 1.5 ^bB^	66.7 ± 8.5 ^bB^
ALG + 0.10% MEL + nZnO	0.047 ± 0.007 ^bA^	5.95 ± 2.84 ^aA^	1.98 ± 1.04 ^aA^	97.5 ± 1.5 ^bA^	129.3 ± 16.6 ^aA^
ALG + 0.25% MEL + nZnO	0.044 ± 0.010 ^aA^	5.45 ± 1.85 ^aA^	1.39 ± 0.67 ^aA^	100.8 ± 1.5 ^cB^	126.2 ± 16.2 ^aA^
ALG + 0.50% MEL + nZnO	0.041 ± 0.008 ^aA^	6.10 ± 3.06 ^aA^	1.60 ± 0.32 ^aA^	96.9 ± 1.5 ^bA^	122.6 ± 15.7 ^aA^
ALG + 0.10% MEL + nAg	0.033 ± 0.003 ^aA^	14.99 ± 3.16 ^bA^	1.69 ± 0.25 ^aA^	105.1 ± 1.6 ^bA^	158.4 ± 20.3 ^bA^
ALG + 0.25% MEL + nAg	0.030 ± 0.006 ^aA^	15.64 ± 8.27 ^bA^	2.19 ± 0.36 ^aA^	95.3 ± 1.4 ^cB^	147.7 ± 18.9 ^bA^
ALG + 0.50% MEL + nAg	0.037 ± 0.012 ^cA^	15.78 ± 6.47 ^bA^	2.40 ± 1.06 ^aA^	97.5 ± 1.5 ^cB^	151.7 ± 19.4 ^bA^

Values are means ± standard deviation. Means with different lowercase (a–c) are significantly different at *p* < 0.05 (compared to control), means with different uppercase (A–C) are significantly different at *p* < 0.05 (compared to other variants within groups).

**Table 4:** Color (L*, a*, b*), total color difference (∆*E*), yellowness index (*YI*) and transmittance of unmodified and modified alginate films.

Sample	L*	a*	b*	∆*E*	*YI*	T_280_ (%)	T_660_ (%)
**ALG**	89.06 ± 0.76 ^a^	−0.64 ± 0.04 ^a^	6.75 ± 1.58 ^a^	Used as standard	11.07 ± 2.84 ^ab^	96.95	97.01
**ALG + 0.10% MEL**	88.87 ± 1.10 ^aA^	−0.62 ± 0.04 ^aA^	6.93 ± 1.67 ^aA^	1.60 ± 1.04 ^A^	11.59 ± 2.82 ^ab^	61.22	87.65
**ALG + 0.25% MEL**	88.21 ± 0.54 ^abA^	−0.60 ± 0.02 ^aA^	8.56 ± 1.03 ^abAB^	2.03 ± 0.77 ^B^	13.84 ± 1.94 ^ab^	62.38	86.73
**ALG + 0.50% MEL**	87.56 ± 2.21 ^bA^	−0.48 ± 0.20 ^bA^	10.19 ± 5.12 ^bB^	4.64 ± 4.70 ^C^	15.48 ± 4.82 ^a^	43.67	85.24
**ALG + 0.10% MEL + nZnO**	87.96 ± 0.57 ^ab^	−0.63 ± 0.03 ^aA^	9.02 ± 1.03 ^bA^	2.37 ± 1.16 ^A^	14.65 ± 1.77 ^ab^	61.19	71.76
**ALG + 0.25% MEL + nZnO**	86.73 ± 1.04 ^b^	−0.44 ± 0.11 ^bB^	11.03 ± 1.58 ^cB^	4.72 ± 1.88 ^B^	18.19 ± 2.82 ^ac^	42.6	68.21
**ALG + 0.50% MEL + nZnO**	84.32 ± 0.84 ^c^	0.07 ± 0.20 ^cC^	15.07 ± 1.38 ^dC^	9.46 ± 1.61 ^C^	25.56 ± 2.61 ^c^	39.39	70.14
**ALG + 0.10% MEL + nAg**	24.94 ± 4.92 ^bA^	24.35 ± 2.81 ^bA^	26.65 ± 6.30 ^bA^	72.21 ± 1.15 ^A^	129.86 ± 17.10 ^b^	24.28	34.07
**ALG + 0.25% MEL + nAg**	24.39 ± 2.53 ^bA^	25.57 ± 2.24 ^bA^	23.60 ± 4.36 ^bcA^	71.94 ± 0.65 ^A^	137.25 ± 11.89 ^c^	10.1	12.57
**ALG + 0.50% MEL + nAg**	22.61 ± 2.99 ^cA^	24.48 ± 4.53 ^bA^	20.94 ± 6.04 ^cA^	72.82 ± 0.68 ^A^	149.47 ± 18.47 ^d^	4.19	10.29

Values are means ± standard deviation. Means with different lowercase (a–d) are significantly different at *p* < 0.05 (compared to control), means with different uppercase (A–C) are significantly different at *p* < 0.05 (compared to other variants within groups).

**Table 5:** Reducing power (RP) and radical (DPPH, ABTS, $O_{2}^{-}$) scavenging activity of unmodified and modified alginate films.

Sample	RP (700 nm)	DPPH (%)	ABTS (%)	$O_{2}^{-}$ (%)
**ALG**	0.023 ± 0.002 ^a^	8.60 ± 0.00 ^a^	7.27 ± 2.39 ^a^	7.47 ± 1.12 ^a^
**ALG + 0.10% MEL**	0.026 ± 0.007 ^aA^	90.62 ± 0.00 ^bA^	90.87 ± 0.02 ^bA^	83.79 ± 3.37 ^bA^
**ALG + 0.25% MEL**	0.027 ± 0.004 ^aA^	89.38 ± 0.02 ^cB^	90.86 ± 0.11 ^bA^	80.50 ± 1.39 ^bA^
**ALG + 0.50% MEL**	0.035 ± 0.011 ^bA^	89.30 ± 0.09 ^dC^	90.78 ± 0.12 ^bA^	81.67 ± 1.21 ^bA^
**ALG + 0.10% MEL + nZnO**	0.016 ± 0.002 ^bA^	5.15 ± 0.00 ^bA^	35.24 ± 0.42 ^bA^	24.02 ± 1.43 ^bA^
**ALG + 0.25% MEL + nZnO**	0.024 ± 0.010 ^aAB^	5.69 ± 0.07 ^cB^	40.39 ± 0.57 ^cB^	47.74 ± 1.17 ^cB^
**ALG + 0.50% MEL + nZnO**	0.025 ± 0.003 ^aB^	6.20 ± 0.00 ^dC^	47.51 ± 0.23 ^dC^	59.25 ± 4.14 ^dC^
**ALG + 0.10% MEL + nAg**	0.030 ± 0.022 ^bA^	0.04 ± 0.00 ^bA^	22.70 ± 0.13 ^bcA^	68.39 ± 3.65 ^bA^
**ALG + 0.25% MEL + nAg**	0.056 ± 0.018 ^cAB^	0.19 ± 0.07 ^cB^	21.56 ± 1.12 ^bB^	68.30 ± 7.78 ^bA^
**ALG + 0.50% MEL + nAg**	0.084 ± 0.046 ^dB^	1.36 ± 0.07 ^dC^	23.92 ± 0.48 ^cC^	78.41 ± 6.33 ^cB^

Values are means ± standard deviation. Means with different lowercase (a–d) are significantly different at *p* < 0.05 (compared to control), means with different uppercase (A–C) are significantly different at *p* < 0.05 (compared to other variants within groups).

**Table 6:** Summary of the size of the zones of growth inhibition (expressed in mm) for *E. coli* (EC) and *S. aureus* (SA) around discs made of films modified with AgNP and ZnONP.

Sample	EC (mm)	SA (mm)
ALG + 0.10% MEL + nZnO	2.53 ± 0.38 ^a^	3.02 ± 0.84 ^a^
ALG + 0.25% MEL + nZnO	3.08 ± 1.21 ^a^	3.08 ± 0.72 ^a^
ALG + 0.50% MEL + nZnO	3.12 ± 0.93 ^a^	3.54 ± 0.66 ^a^
ALG + 0.10% MEL + nAg	1.44 ± 0.47 ^b^	2.53 ± 0.54 ^a^
ALG + 0.25% MEL + nAg	2.59 ± 0.79 ^ab^	3.08 ± 1.12 ^a^
ALG + 0.50% MEL + nAg	2.74 ± 0.41 ^a^	3.52 ± 0.60 ^a^

Values are means ± standard deviation. Means with a different letters (a-b) are significantly different at *p* < 0.05 (compared to other variants within groups).

## Supplementary Materials

The following are available online at https://www.mdpi.com/article/10.3390/ma15072381/s1, Figure S1: Inverted light microscopy of L929 murine fibroblasts, Figure S2: Inverted light microscopy of L929 murine fibroblasts incubated for 24 h, Table S1: Example photographs of film samples and the inhibition zones they produced against E. coli and S. aureus microorganisms.
