# Supplementary material for: Alginate Biofunctional Films Modified with Melanin from Watermelon Seeds and Zinc Oxide/Silver Nanoparticles
Source: Materials (Basel). 2022 Mar 23;15(7):2381. doi: 10.3390/ma15072381 (PMC8999530; doi:10.3390/ma15072381)
Supplement: Supplementary file 1 [file materials-15-02381-s001.zip › materials-1623218-supplementary.pdf]

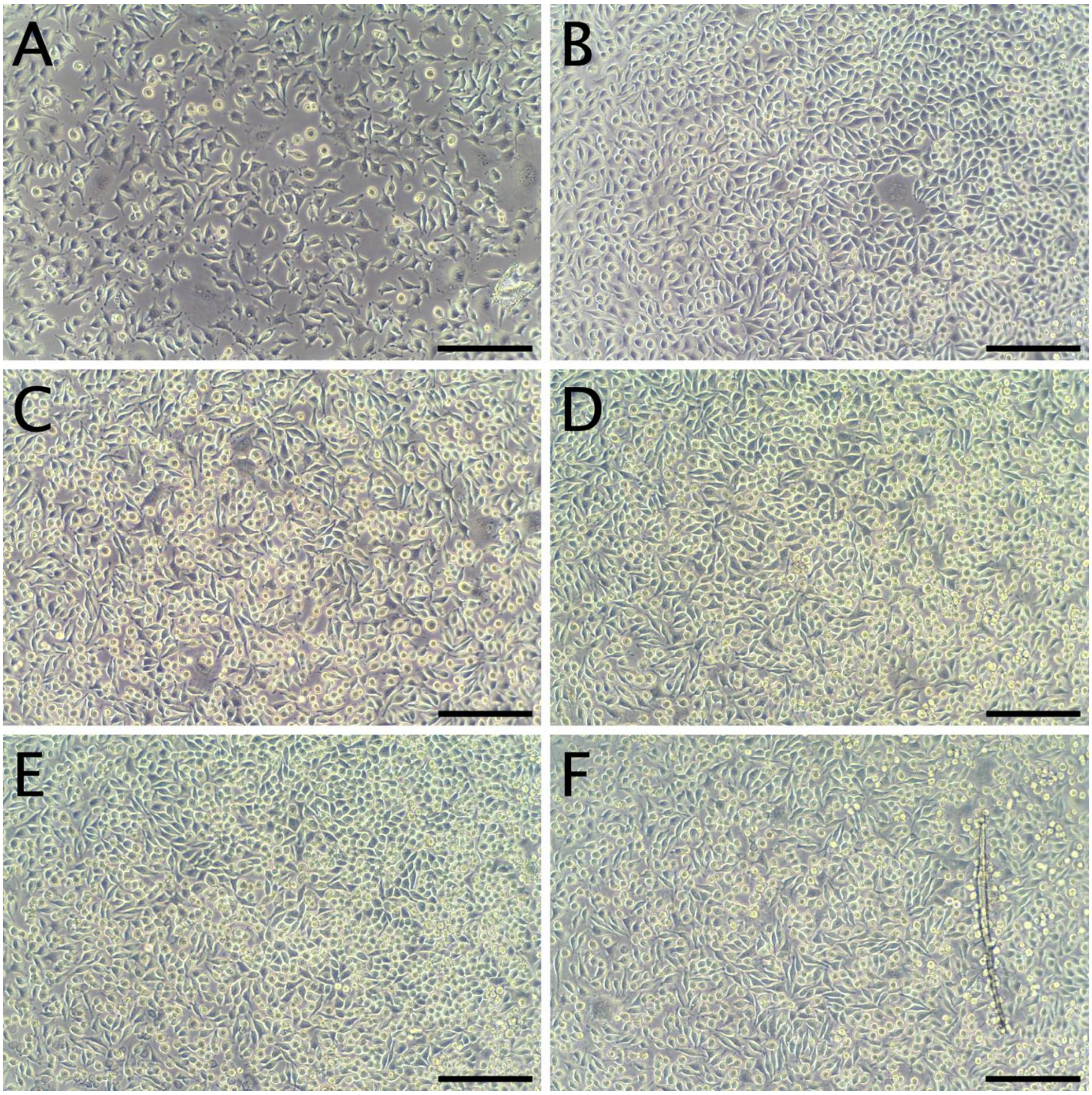

Figure S1. Inverted light microscopy of L929 murine fibroblasts. (**A**) 24 hours after seeding. (**B**) 24 hours after sham incubation. (**C**) 24 hours after incubation with alginate film (control), (**D**) 24 hours after incubation with Alg-0.1% MEL film, (**E**) 24 hours after incubation with Alg-0.25% MEL film, (**F**) 24 hours after incubation with Alg-0.5% MEL film.

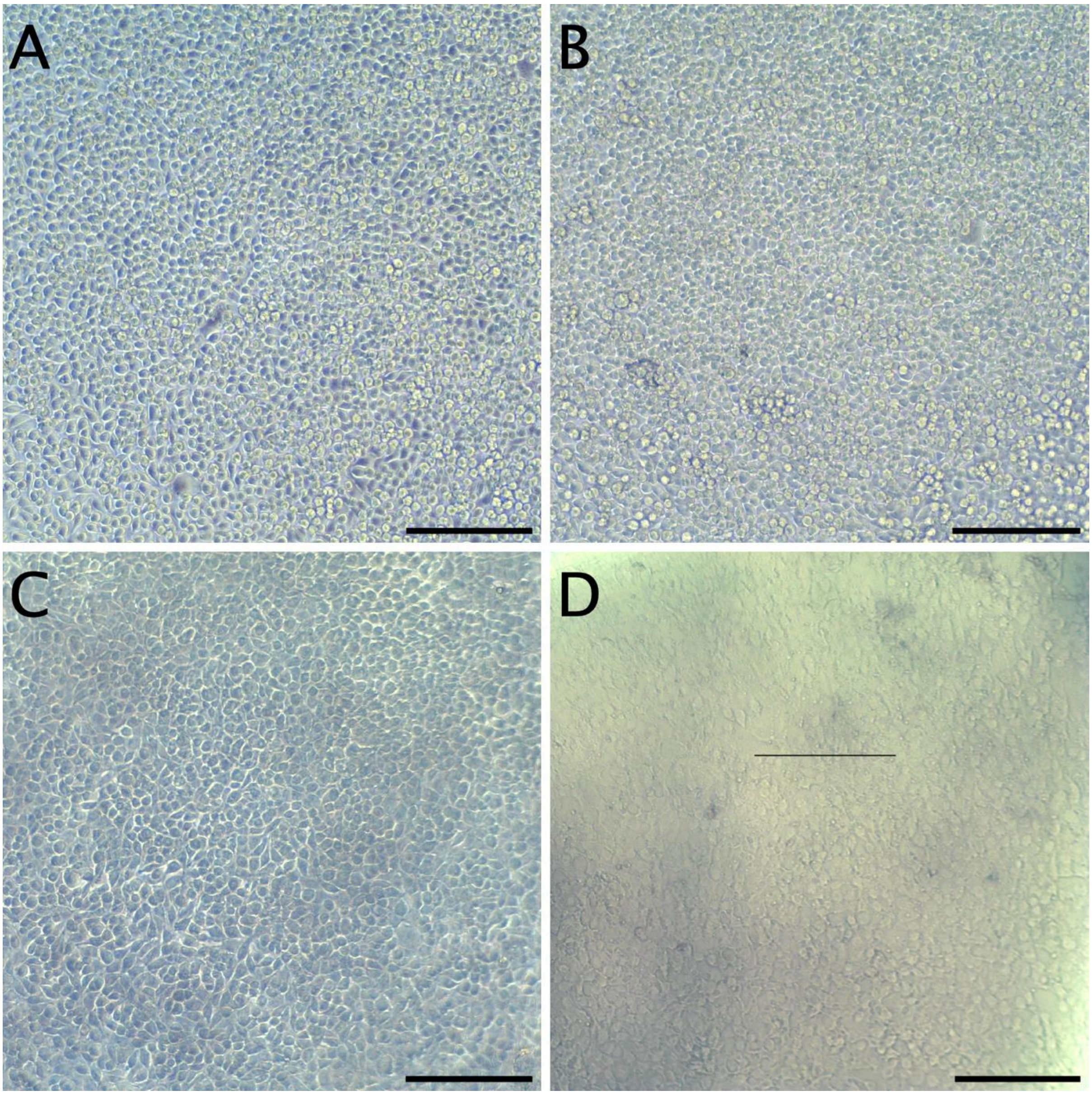

Figure S2. Inverted light microscopy of L929 murine fibroblasts incubated for 24 hours with: (**A**) sham (tissue culture plastic only), (**B**) Alg-0.5% MEL film, (**C**) Alg-ZnONP-0.5% MEL film, and (**D**) Alg-AgNP-0.5% MEL film.

Sample	EC	SA
ALG + 0.50% MEL	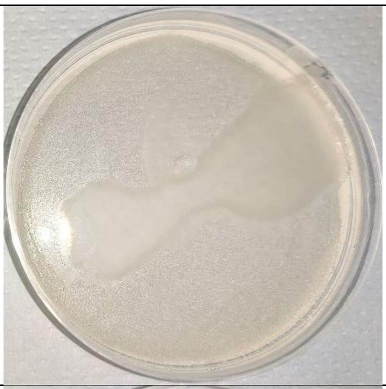	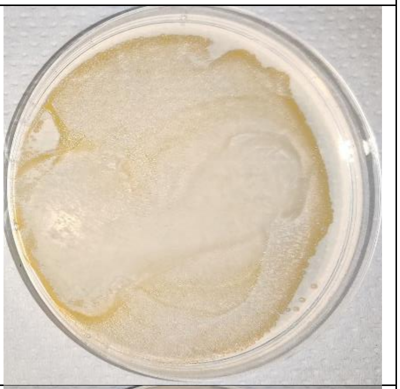
ALG + 0.50% MEL + nZnO	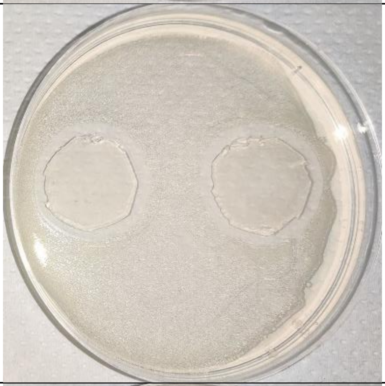	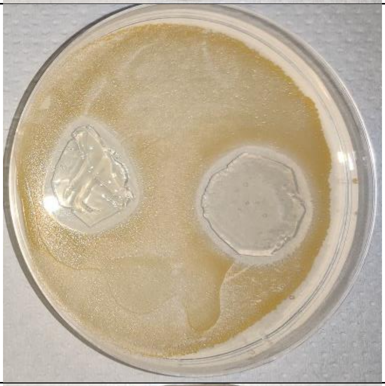
ALG + 0.50% MEL + nAg	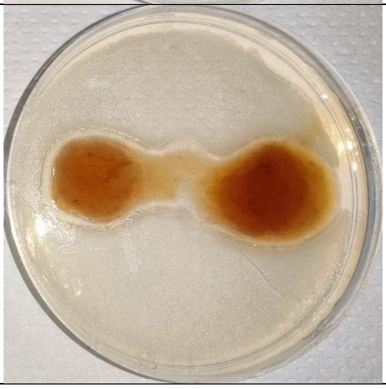	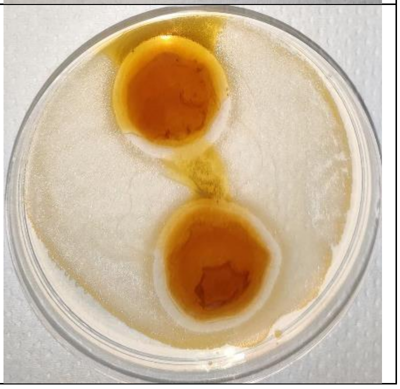

Table S1. Representative photographs of film samples and the growth inhibition zones against E. coli and S. aureus.
